# A second contender for “world’s smallest fly” (Diptera: Phoridae)

**DOI:** 10.3897/BDJ.6.e22396

**Published:** 2018-01-24

**Authors:** Brian V. Brown

**Affiliations:** 1 Natural History Museum of Los Angeles County, Los Angeles, United States of America

**Keywords:** tropical, parasitoid, biodiversity, taxonomy

## Abstract

**Background:**

Flies of the family Phoridae (Insecta: Diptera) are amongst the most diverse insects in the world, with an incredible array of species, structures and life histories. Wiithin their structural diversity is the world's smallest fly, *Euryplatea nanaknihali* Brown, 2012.

**New information:**

A second minute, limuloid female phorid parasitoid fly (Diptera: Phoridae) is described. Known from a single specimen from a site near Manaus, Brazil, *Megapropodiphora arnoldi* **gen. n., sp. n.** is only 0.395 mm in body length, slightly smaller than the currently recognised smallest fly, *Euryplatea nanaknihali* from Thailand. The distinctive body shape of *M. arnoldi*, particularly the relatively enormous head, mesothorax and scutellum, the latter of which covers most of the abdomen, easily separates it from other described phorids. Most remarkably, the forelegs are extremely enlarged, whereas mid- and hind legs are reduced to small, possibly vestigial remnants. A possible male specimen, unfortunately destroyed during processing, is briefly described.

## Introduction

There are few other families of insects with such a wide variety of body forms and life histories as the Phoridae, humpbacked or scuttle flies. Particularly in tropical forests, there is always the possibility of discovering a new, jaw-droppingly bizarre species, or one that has incredibly specialised food and mate acquisition behaviour. Phorids are found nearly everywhere on earth, in faunas of seemingly endless numbers of species. They have such a litany of modified body parts and have conquered such a wide range of food sources that we are running out of superlatives to characterise the family.

**Figure 1.**
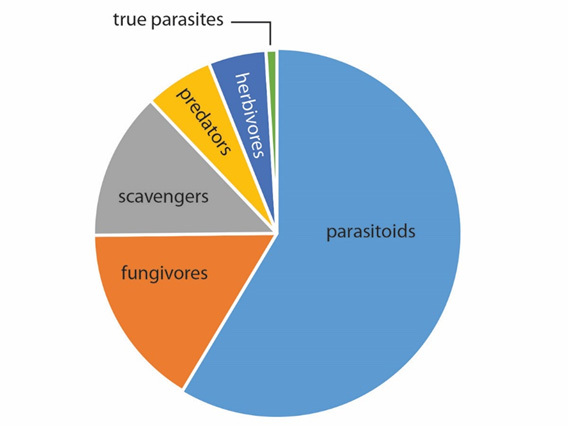
Known lifestyles of Phoridae (data from Disney 1994).

Although phorids are commonly described as scavengers, with a few parasitoid species, this generalisation is false. The “scavenger” moniker commonly affixed to phorids is based largely on the ubiquity and abundance of *Megaselia scalaris* (Loew, 1866), a cosmopolitan, synanthropic and highly polyphagous species (Disney 2008). Based on our current knowledge, phorids are in fact mostly parasitoids (Fig. 1). They comprise the third largest group of parasitoid Diptera, after Tachinidae and Bombyliidae. Lifestyles of most phorid species are still unknown, however.

Previously, one of the most unusual and remarkable phorids ever discovered was described, the minute (0.4 mm body length) female of *Euryplatea nanaknihali* from Thailand (Brown 2012). Although known from a single female, the fly displayed characters of the African, ant-parasitoid genus *Euryplatea* Schmitz, 1941, such as the limuloid body form and a dark coloured, triangular wing rudiment. Furthermore, based on the shape of its oviscape, it was clearly a parasitoid and one that was capable of developing in the head capsule of some of the smallest ants in the world. The fly described herein is another extremely modified, extremely small female phorid that is even more unusual than *E. nanaknihali* and is from the New World tropics.

## Materials and methods

The specimen described herein was collected by a Malaise trap (Townes 1972) in a tropical forest near Novo Airão, Amazonas, Brazil (2.71°S, 60.95°W). It was slide-mounted whole in Canada balsam after dehydration in 95% ethanol and clearing in clove oil.

## Taxon treatments

### Megapropodiphora

Brown, 2018 gen. n.

urn:lsid:zoobank.org:act:AC55B401-F579-4024-91B0-39D8E204A5F5

Megapropodiphora Megapropodiphora arnoldi Brown 2018 Status: new species described in this paper.

#### Description

Same as for species.

#### Diagnosis

There are a small number of minute, limuloid phorid genera in the world. In the New World tropics, the only relatively similar genera have large, differentiated frontal setae that are several times longer than the short frontal setae and do not have the scutellum covering the abdomen (Brown 1993). The Old World species of the genus *Euryplatea* Schmitz, likewise differ by having the abdomen not covered by the scutellum and by having a solid, triangular wing rudiment (Brown 2012).

### Megapropodiphora arnoldi

Brown 2018 sp. n.

urn:lsid:zoobank.org:act:5520DF82-73A3-40EC-8619-A05157C51F59

#### Materials

- **Type status:** Holotype. **Occurrence:** recordedBy: D. Amorim, J. Raphael; sex: female; lifeStage: adult; preparations: mounted on slide in Canada Balsam by B. Browns; **Taxon:** genus: Megapropodiphora; specificEpithet: arnoldi; scientificNameAuthorship: Brown 2018; **Location:** country: Brazil; stateProvince: Amazonas; locality: 12 km S Novo Airão; verbatimElevation: 34 m; locationRemarks: forest; decimalLatitude: -02.71; decimalLongitude: -60.95; **Event:** samplingProtocol: Malaise trap; eventDate: 2013-12-08/2013-12-09
- **Type status:** Holotype. **Occurrence:** otherCatalogNumbers: LACM ENT 334268

#### Description

**Figure 2.**
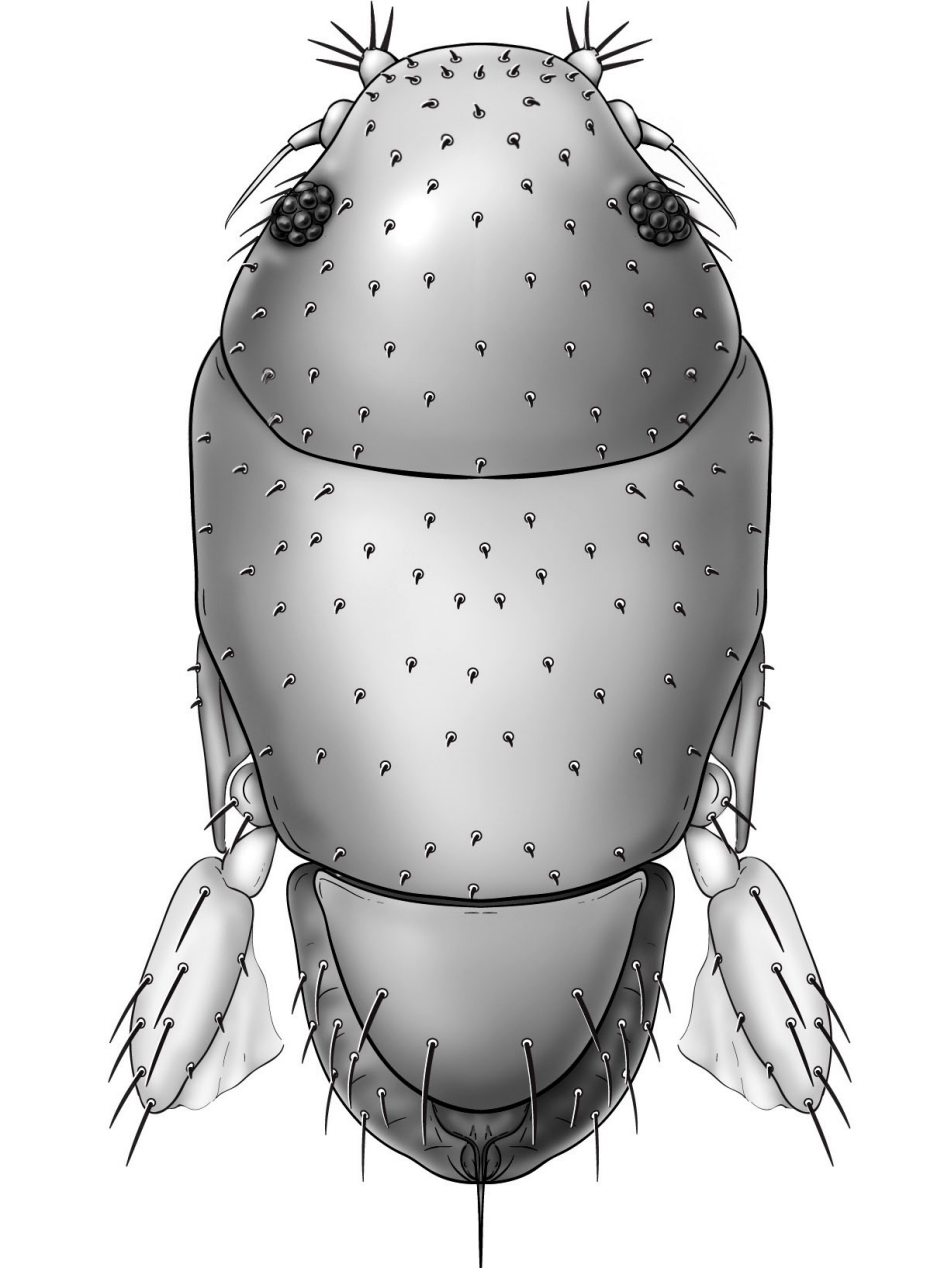
*Megapropodiphora arnoldi* **sp. n.**, female, dorsal view. Body length=0.395 mm. Drawing by I. Strazhnik.

**Figure 3.**
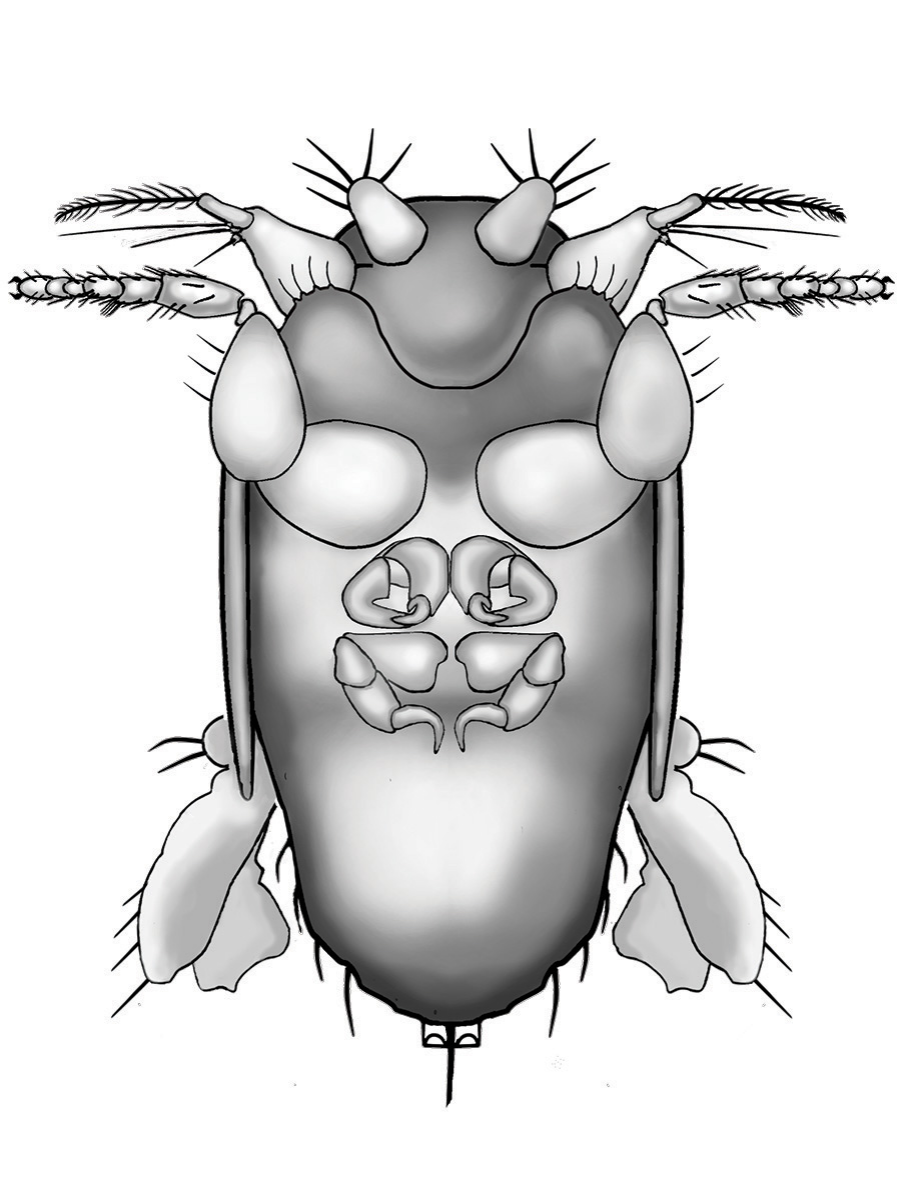
*Megapropodiphora arnoldi* **sp. n.**, female, ventral view. Legs re-arranged for easier viewing. Structure of mid- and hind legs approximate. Drawing by T. Hayden.

Female (Figs 2, 3). Body length 0.395 mm, flattened, limuoid. Flagellomere 1 pointed, with long setae almost as long as flagellomere 1 + apparently 2-articled arista (difficult to discern due to small size of specimen). Palpus large, broad with apical setae. Proboscis greatly reduced. Genal margin with few setae. Frons broad, without differentiated bristle-like setae; eye greatly reduced to few ommatidia. Scutum with short sparse setae dorsally. Scutellum with 2 pairs of large setae. Forelegs, especially forecoxa, greatly enlarged; forefemur with three anteroventral setae. Mid- and hind legs greatly reduced, possibly vestigial. Abdomen extremely small, covered by scutellum. Oviscape pointed.

Male unknown (but see below).

#### Diagnosis

**Figure 4.**
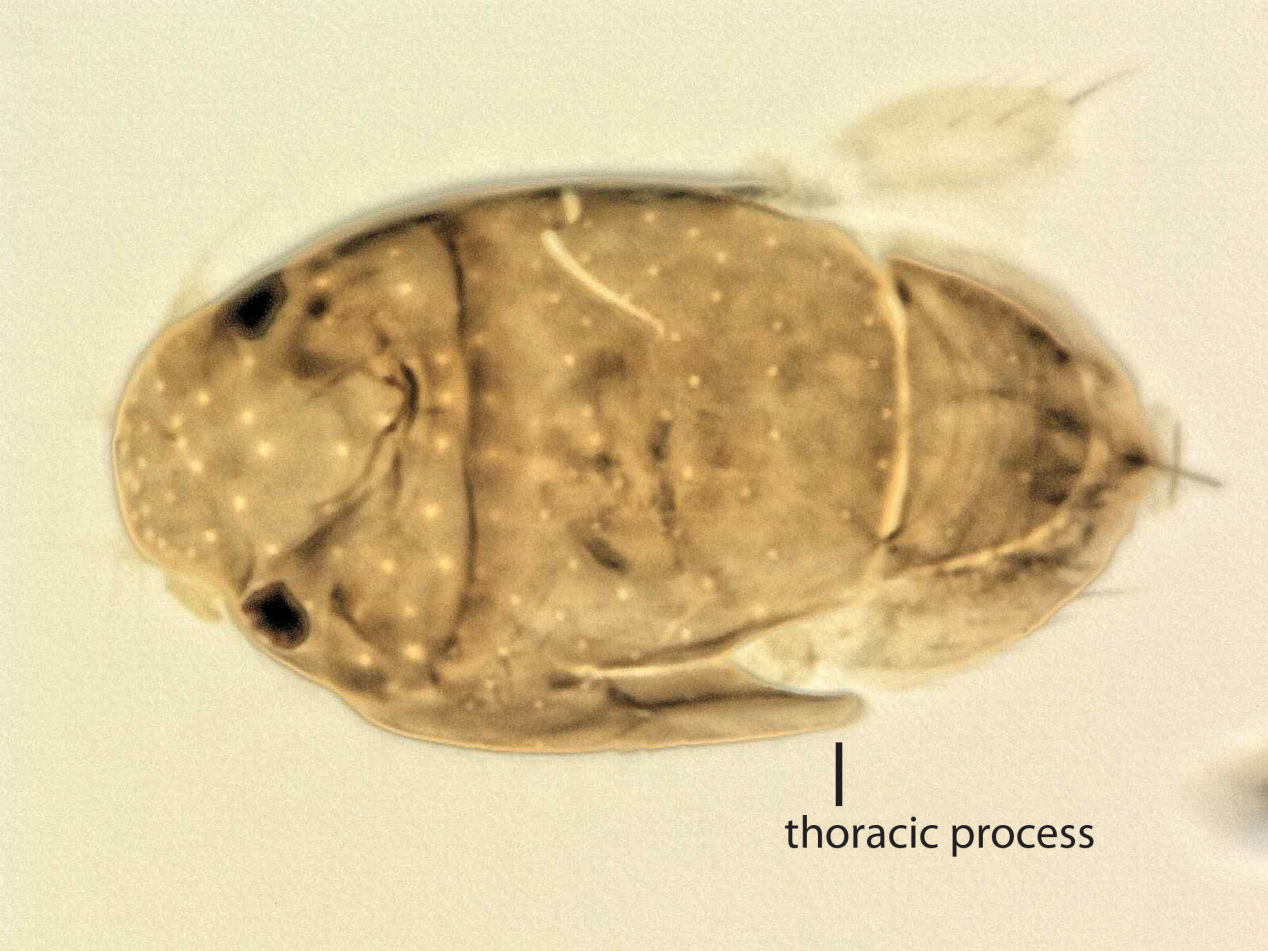
*Megapropodiphora arnoldi* **sp. n.**, female, dorsal, slightly oblique. Photomicrograph by B. Brown.

Female. Minute, limuloid; body setae scattered, sparse; wing with shed blades and short costa; head and scutum large, scutellum covering almost entire abdomen; oviscape thin, pointed, indicating a parasitoid lifestyle. Edge of scutum lateroventrally extended, posteriorly ending in narrowed flange (Fig. 4). Forelegs greatly enlarged; mid- and hind legs reduced.

Similar genera. Males of *Brachycosta* Prado, 1976, have a short costa, but much longer than that of *Megapropodiphora* gen. n., are much larger in size and have a larger frons and head. Females of this new genus are differentiated from all other phorids by minute size, leg structure and elongation of the scutellum to cover the abdomen.

#### Etymology

The genus name is Latin for large foreleg, referring to the structure of the female. The specific epithet refers to Arnold Schwarzenegger, former governor of California, whose own greatly enlarged forelimbs distinguished him in his pre-political careers.

#### Distribution

Amazonian Brazil

#### Biology

Unknown, but almost certainly a parasitoid. The torn wing membrane is reminiscent of other phorid flies that shed their wings when entering a social insect colony. It seems likely that the greatly enlarged forelegs are used to clutch a host, upon which the small, rounded body would appear similar to that of many phoretic mites.

#### Notes

A potential male specimen was accidentally destroyed during illustration process, but from memory only, it was as follows: minute, with small head; frons greatly reduced (as in male *Chonocephalus* Wandolleck, 1989) and extremely reduced head setae; wing with short costa and large blade. Lacking further information, I cannot assign this new genus and species to any subfamily.

It is common for researchers to change the alcohol in Malaise trap samples, pouring off the old liquid, stained yellow with body fluids of the many preserved insects. Also, we commonly drain the alcohol out of samples for safer or at least more legal transport of these chemicals. Fortunately, the samples including the *M. arnoldi* sp. n. were examined immediately after being collected and were thus not so treated. The tiny phorid flies described herein are easily lost when waste alcohol is drained off, such that any alcohol that is being disposed should be first examined carefully under a microscope. Furthermore, I suggest purposely “washing” Malaise trap samples in fresh alcohol to clean tiny insects off of the larger ones and search for further microfauna.

## Supplementary Material

XML Treatment for Megapropodiphora

XML Treatment for Megapropodiphora arnoldi
